# Classification of fall risk in Parkinson’s disease using empirical mode decomposition and machine learning

**DOI:** 10.3389/fbioe.2026.1790452

**Published:** 2026-03-19

**Authors:** Yunfei Gui, Yuyi Fan, Linyan Liu, Jinfeng Cao, Linlin Zhang, Yi Yu, Roger Adams, Jia Han, Jie Lyu

**Affiliations:** 1 School of Health Science and Engineering, University of Shanghai for Science and Technology, Shanghai, China; 2 Department of Orthopedics, Jinshan District Central Hospital Affiliated to Shanghai University of Medicine and Health Sciences, Shanghai, China; 3 College of Medical Instruments, Shanghai University of Medicine and Health Sciences, Shanghai, China; 4 Periodicals Agency of Shanghai University, Shanghai University, Shanghai, China; 5 College of Rehabilitation Sciences, Shanghai University of Medicine and Health Sciences, Shanghai, China; 6 Research Institute for Sport and Exercise, University of Canberra, Canberra, ACT, Australia

**Keywords:** center of pressure, empirical mode decomposition, fall risk classification, machine learning, Parkinson’s disease

## Abstract

**Introduction:**

Fall risk assessment in Parkinson’s Disease (PD) is critical for preventing patient injuries; however, traditional clinical scales are often constrained by their semi-quantitative nature and time-consuming administration. This study aimed to develop and validate an analytical framework integrating Empirical Mode Decomposition (EMD) and machine learning for the objective classification of fall risk in PD patients.

**Methods:**

Data from 32 PD patients under four standing conditions were analyzed, with fall risk labels defined based on a Mini-BESTest score cutoff of ≤21. Center of Pressure (COP) signals were first decomposed into Intrinsic Mode Functions (IMFs) using EMD, from which multi-dimensional features were extracted. Subsequently, the Sequential Forward Floating Selection (SFFS) algorithm was employed to identify a core feature subset, and the performance of five classifiers—Random Forest (RF), Decision Tree (DT), Extreme Gradient Boosting (XGB), Logistic Regression (LR), and Support Vector Machine (SVM)—was evaluated using Leave-One-Subject-Out Cross-Validation (LOSO-CV). Finally, a multi-trial probability aggregation strategy was introduced to assess subject-level risk.

**Results:**

Results indicated that the rigid surface eyes-closed (rs ec) condition yielded the optimal discriminatory capability. The SFFS algorithm identified a parsimonious 3-dimensional feature subset (COPap_IMF1_BinEntropy, COPml_IMF1_STD, COPap_IMF2_TotalPower), which enabled tree-based classifiers to exhibit superior performance. Notably, DT and RF achieved highly balanced sensitivity and specificity, reaching a maximum subject-level AUC of 0.96.

**Discussion:**

In conclusion, EMD-derived multi-scale features can precisely capture postural control deficits in PD, offering a promising technical solution for the future development of efficient and objective PD clinical monitoring tools.

## 1 Introduction

In terms of global burden, Parkinson’s Disease (PD) is a fast-growing neurodegenerative disorder, with the number of individuals affected worldwide increasing from 2.5 million in 1990 to 6.1 million in 2016, and impacting approximately 2%–3% of the population over 65 years of age (Poewe et al., 2017; Dorsey et al., 2018). The core pathological hallmark of PD is the loss of dopaminergic neurons in the substantia nigra, leading to a dopamine deficiency in the striatum. This manifests clinically through a set of classic motor signs, including resting tremor, bradykinesia, muscle rigidity, and the loss of postural reflexes (Jankovic, 2008). Among these symptoms, postural instability is a cardinal feature of PD and a primary contributor to associated functional limitations and falls (Wood et al., 2002; Bloem et al., 2004).

Postural instability significantly increases the fall risk in patients with PD. It is estimated that approximately 60% of PD patients experience at least one fall, with nearly 40% experiencing recurrent falls (Pickering et al., 2007; Allen et al., 2013). These fall events can lead to fractures, soft tissue injuries, hospitalization, and even death (Hely et al., 2005; Williams et al., 2006; Contreras and Grandas, 2012; Pouwels et al., 2013). Consequently, falls are a critical factor that limits the independent living ability of PD patients and may negatively affect their life expectancy (Fasano et al., 2017). This cascade of problems not only imposes a heavy physical and psychological burden on patients and their families, but also results in socioeconomic pressure. In the United States alone, the total annual economic burden associated with PD exceeded $14.4 billion in 2010 (Kowal et al., 2013).

Currently, the clinical assessment of fall risk in PD patients relies on several well-established clinical scales, such as the motor examination section of the Unified Parkinson’s Disease Rating Scale (UPDRS) (Goetz et al., 2008), the Hoehn & Yahr (H&Y) stage (Hoehn and Yahr, 1967), the Falls Efficacy Scale-International (FES-I) (Yardley et al., 2005). While these tools provide an important basis for clinical decision-making, it is well-recognized that their semi-quantitative nature, potential for ceiling effects, and limited responsiveness can restrict the precise and objective assessment of postural instability (Mancini and Horak, 2010; Winser et al., 2019). Therefore, developing an objective and quantitative assessment tool for more accurate fall risk classification in PD patients is crucial (Hubble et al., 2015).

In this context, force platform-based posturography, particularly the analysis of the dynamic characteristics of the Center of Pressure (COP) trajectory during quiet standing, has emerged as a significant research instrument (Prieto et al., 1996; Chen et al., 2021). The COP, as a comprehensive reflection of the continuous regulatory activity of the neuromuscular system during balance maintenance, contains time-series data that can reveal subtle impairments in balance control mechanisms (Winter, 1995; Palmieri et al., 2002). However, the raw COP signal is a complex, non-linear, and non-stationary time series (Błaszczyk, 2016), and traditional linear parameters extracted may fail to fully capture its dynamic properties (Duarte and Freitas, 2010).

While machine learning algorithms have shown promise in assessing fall risk—as further evidenced by recent studies confirming the feasibility of kinematic data for long-term risk classification (Sotirakis et al., 2024) and validating the robustness of machine learning models in multicenter clinical applications (Malaguti et al., 2025)—their performance depends heavily on the quality of feature extraction. To better utilise the information in COP signals, this study sought to explore the validity of a signal processing and data analysis method based on Empirical Mode Decomposition (EMD) for classifying fall risk in PD patients. As a powerful, adaptive, time-frequency analysis technique, EMD can decompose a complex signal into a series of Intrinsic Mode Functions (IMFs), each representing a different time-scale characteristic. Its primary advantage over other methods, such as wavelet transforms, is its fully data-driven nature, which does not require a pre-determined basis function, making it particularly suitable for analyzing complex physiological signals like COP. EMD has shown potential in processing non-linear physiological signals (Huang et al., 1998; Pachori et al., 2008; Zeng et al., 2019). Extracting multi-dimensional features from these IMFs and combining them with Machine Learning techniques to build models for risk classification may potentially characterize the postural control of PD patients more comprehensively and accurately, and achieve more reliable identification of fall risk (Gao et al., 2018; Moon et al., 2020). Therefore, the primary objective of this study was to develop and validate a machine learning model, utilizing features derived from EMD of COP signals, to classify fall risk status in patients with PD, and thus provide useful information for timely clinical intervention.

## 2 Materials and methods

### 2.1 Dataset preparation

The dataset was prepared from 32 idiopathic PD patients (male/female: 24/8) aged between 44 and 81 years, with a body-mass index ranging from 17.5 to 31.5 kg/m^2^. All data were sourced from a publicly available, anonymized posturography repository (de Oliveira et al., 2022); thus, additional ethical review was waived for this secondary analysis. Based on the original cohort description, the participants were not *de novo* patients; rather, they were individuals undergoing stable dopaminergic therapy for at least 1 month, with an average disease duration of 7.23 (±4.49) years and disease severities spanning Hoehn & Yahr stages 1–4. To eliminate the acute confounding effects of medication on motor presentation, only data acquired during the off-medication state (i.e., at least 12 h after the last dose) were included in this study.

The data acquisition protocol involved static quiet standing assessments under four sensory conditions: rigid surface with eyes open, rigid surface with eyes closed, unstable (foam) surface with eyes open, and unstable surface with eyes closed. Each task was maintained for 30 s and repeated three times. During the trials, an AMTI OPT400600-1000 force plate continuously recorded three-dimensional ground reaction forces and moments at a sampling frequency of 100 Hz. From these kinetic measurements, the COP trajectories in both the anterior-posterior (AP) and medio-lateral (ML) directions were computed. Prior to further analysis, the raw COP signals were smoothed using a fourth-order, zero-phase Butterworth low-pass filter with a cutoff frequency of 10 Hz.

Given that the original public dataset lacked specific records of fall events, the Mini-BESTest score was adopted as a surrogate label for fall risk (Leddy et al., 2011; Duncan et al., 2013). Based on established cutoff criteria for discriminating fall risk in PD reported in the literature (Leddy et al., 2011; Duncan et al., 2013), a threshold of 21 was selected. Under this configuration, the sample distribution consisted of 8 subjects in the Fall Risk group and 24 subjects in the No Fall Risk group. Accordingly, subjects with a Mini-BESTest score of ≤21 were labeled as Fall Risk, while those with a score >21 were labeled as No Fall Risk.

### 2.2 Feature extraction

A series of features was extracted from the COP time-series signals in both the AP and ML directions. A comprehensive list of the 13 extracted features across five domains is provided in Table 1. First, EMD was applied to the COP signal in each direction, decomposing it into a series of IMFs, from IMF1 to IMF5. The selection of the first five IMFs was based on considerations of decomposition stability and feature consistency. Given the 30-s duration of the COP signals in this study, IMF1 through IMF5 could be stably and consistently extracted across all subjects, whereas higher-order components appeared only in a minority of samples. Furthermore, experimental observations indicated that these higher-order components predominantly represented very low-frequency baseline drifts or quasi-periodic residual waves. Subsequently, for each IMF component in each direction, features covering five domains were calculated: time-domain features included Mean, Standard Deviation (STD), Root Mean Square (RMS), Minimum, Maximum, and Interquartile Range (IQR); the frequency-domain feature was Total Power; the relative variability feature was the Coefficient of Variation (CV); non-linear dynamics features were Approximate Entropy (ApEn) and Sample Entropy (SampEn) (Pincus, 1991; Richman and Moorman, 2000); and distributional characteristics included Skewness, Kurtosis, and Bin-Entropy (calculated from a 10-bin histogram). The Bin-Entropy was calculated as follows:

$$Bin-Entropy=-\sum_{i=1}^{10}p_{i}\ln\left(p_{i}\right)$$

where i is the bin index, and $p_{i}$ represents the probability of the signal amplitude falling within the i -th bin.

**TABLE 1:** Comprehensive list of multi-domain features extracted from COP signals and their IMFs.

Feature category	Feature name
Time-domain	Mean
	Standard deviation, STD
	Root mean square, RMS
	Minimum
	Maximum
	Interquartile range, IQR
Frequency-domain	Total power
Relative variability	Coefficient of variation, CV
Non-linear complexity	Approximate entropy, ApEn
	Sample entropy, SampEn
Distributional characteristics	Skewness
	Kurtosis
	Bin-entropy

In total, 384 trial-level samples (32 subjects × 4 conditions × 3 trials) were processed to construct the initial feature dataset. Through this process, a comprehensive multi-dimensional feature vector containing 130 features (2 directions × 5 IMFs × 13 features) was constructed for each trial record, which was used for subsequent machine learning modeling.

To systematically manage the large number of extracted features, a three-part naming convention was adopted. The first part specifies the signal source, either the ML COP (COPml) or the AP COP (COPap). The second part defines the signal component, specifically one of the five IMFs (IMF1 to IMF5) obtained via EMD. The final part represents the specific feature metric, such as Mean or SampEn. For example, a feature named COPap_IMF3_CV represents the CV of the third IMF component derived from the EMD of the AP COP signal.

### 2.3 Model construction and validation

In this study, the Random Forest (RF) algorithm was selected as the baseline model for classifying fall risk in patients with PD. RF was chosen because, as an ensemble learning method, it leverages the collaborative decision-making of multiple decision trees to effectively mitigate the risk of overfitting in small-sample scenarios while capturing non-linear associations between biomechanical features and fall risk. Concurrently, four other classic algorithms—Decision Tree (DT), Extreme Gradient Boosting (XGB), Logistic Regression (LR), and Support Vector Machine (SVM)—were included for performance comparison to comprehensively evaluate the applicability and discriminatory power of different models for this task. To identify the signal acquisition scenario that best exposes postural control deficits, a systematic evaluation was conducted across all candidate standing conditions (rigid surface eyes-open, rigid surface eyes-closed, unstable surface eyes-open, unstable surface eyes-closed, and all conditions combined) using a unified modeling framework and validation protocol. Subsequent feature selection and model optimization proceeded based on the optimal condition identified from this evaluation. To address the issues of model redundancy and overfitting caused by the high-dimensional feature space, the Sequential Forward Floating Selection (SFFS) algorithm was employed for core feature screening (Pudil et al., 1994). This process was strictly nested within the cross-validation framework and relied solely on training data. The objective was to maximize the Area Under the Receiver Operating Characteristic Curve (ROC) of the baseline model by iteratively incorporating features with the optimal discriminatory contribution until no further effective performance gain was observed. This approach prevented information leakage from the test set, thereby ensuring the generalization reliability of the feature subset. All models were trained and evaluated using Leave-One-Subject-Out Cross-Validation (LOSO-CV) (Saeb et al., 2017). In each iteration, all samples from one subject were designated as the independent test set, while samples from the remaining subjects served as the training set, thus avoiding the issue of pseudo-replication where samples from the same subject appear in both sets. During the training phase, features were standardized to eliminate dimensional discrepancies, and class weights were applied to mitigate learning bias resulting from the imbalance between high and low fall risk groups. The test set was processed using the same standardization parameters as the training set to ensure consistent data distribution. Finally, the final aggregated probability for each subject was calculated by arithmetically averaging the model’s classification probabilities from each independent trial. The optimal classification threshold for each model was determined based on the maximization of the Youden Index (Youden, 1950), balancing discriminatory precision with clinical applicability.

### 2.4 Statistical analysis

Multi-dimensional statistical methods were employed to test inter-group differences, evaluate model performance, and verify result robustness. All statistical inferences were conducted using each subject as an independent observational unit. First, the differences in biomechanical features between Fall Risk and No Fall Risk groups were analyzed. The Shapiro-Wilk test was used to assess the normality of each feature distribution. Features satisfying the normality assumption were analyzed using Welch’s independent samples t-test, while those with non-normal distributions were analyzed using the non-parametric Mann-Whitney U test. The significance level was set at p < 0.05. Second, the subject-level Area Under the Curve (AUC) was utilized as the core metric for model discriminatory capability, alongside sensitivity and specificity to reflect clinical classification efficacy. To ensure statistical reliability, a Bootstrap resampling method with 1,000 iterations was adopted (Efron and Tibshirani, 1994) to calculate the mean, standard deviation, and 95% confidence intervals for the AUC of each model, as well as the expected means for sensitivity and specificity. Beyond discriminatory performance, calibration plots were employed to assess the reliability of predicted probabilities across a continuous range of decision thresholds (Van Calster et al., 2019). Given the limited sample size (N = 32), a quantile binning strategy with three bins was utilized to construct the calibration curves, ensuring a balanced distribution of subjects within each probability interval and mitigating potential statistical fluctuations arising from data sparsity. Furthermore, the Brier score was calculated to quantify calibration accuracy (Rufibach, 2010), where a lower score indicates higher concordance between the predicted probabilities and actual fall risk labels. Finally, with Random Forest serving as the baseline model, the distribution of AUC differences between models was constructed using 1,000 Bootstrap resamples. The significance p-value was calculated via non-parametric Bootstrap hypothesis testing (Efron and Tibshirani, 1994) to evaluate whether significant differences in discriminatory efficacy existed between each model and the baseline.

## 3 Results

Initially, the classification performance across different standing conditions—rigid surface eyes-open (rs eo), rigid surface eyes-closed (rs ec), unstable surface eyes-open (us eo), unstable surface eyes-closed (us ec), and all conditions combined (all)—was systematically evaluated using the Random Forest (RF) baseline model. As presented in Table 2, the rs ec condition yielded the optimal discriminatory capability, achieving a mean AUC of 0.8244 ± 0.0820. The 95% confidence interval (CI) for this condition was [0.6510, 0.9630], with the lower bound exceeding 0.6, indicating robust statistical stability. In comparison, the AUCs for rs eo (0.7519 ± 0.1149) and the combined condition (0.7439 ± 0.1002) were lower. The two unstable surface conditions demonstrated substantial performance volatility (us ec: 0.6189 ± 0.1245; us eo: 0.5223 ± 0.1308), with lower CI bounds falling below 0.5, suggesting unreliable discrimination. Consequently, based on both discriminatory performance and stability, the rs ec condition was selected for all subsequent analyses.

**TABLE 2:** Classification performance of the baseline model for evaluating different signal acquisition scenarios.

Condition	AUC	95% CI
rs ec	0.8244 ± 0.0820	[0.6510, 0.9630]
rs eo	0.7519 ± 0.1149	[0.4827, 0.9566]
all	0.7439 ± 0.1002	[0.5362, 0.9257]
us ec	0.6189 ± 0.1245	[0.3555, 0.8334]
us eo	0.5223 ± 0.1308	[0.2679, 0.7756]

Data are reported as mean ± standard deviation; CI: confidence interval.

Focusing on the rs ec condition, a subject-level statistical analysis was conducted on the initial 130-dimensional feature set to examine differences between Fall Risk and No Fall Risk groups. Eleven features were identified that exhibited statistically significant differences (Table 3). Notably, the Interquartile Range of the first Intrinsic Mode Function in the anterior-posterior direction (COPap_IMF1_IQR) showed the most significant difference (p = 0.0006). Significant group differences were also observed across distributional features (Bin-Entropy, Kurtosis, Skewness), time-domain features (STD, RMS, CV), and frequency-domain features (Total Power) within various IMF bands.

**TABLE 3:** Biomechanical features showing statistically significant differences between No Fall Risk and Fall Risk groups under the rs ec condition.

Feature	Fall Risk	No Fall Risk	p value
COPap_IMF1_IQR	0.3379 ± 0.1133	0.7960 ± 0.5262	0.0006***
COPml_IMF3_CV	22.4702 ± 5.7626	74.5976 ± 59.3361	0.0008***
COPml_IMF4_Kurt	−0.2569 ± 0.2143	0.2107 ± 0.7154	0.0099**
COPap_IMF1_BinEntropy	1.3989 ± 0.2002	1.6621 ± 0.2096	0.0108*
COPml_IMF1_TotalPower	0.0153 ± 0.0077	0.0977 ± 0.1766	0.0154*
COPap_IMF1_Kurt	5.2944 ± 2.4767	2.4353 ± 2.9434	0.0154*
COPml_IMF1_STD	0.1167 ± 0.0329	0.2443 ± 0.1853	0.0176*
COPml_IMF1_RMS	0.1167 ± 0.0329	0.2443 ± 0.1853	0.0176*
COPap_IMF2_Skew	−0.0811 ± 0.1362	0.0604 ± 0.1676	0.0392*
COPap_IMF1_STD	0.4771 ± 0.2279	0.7712 ± 0.5019	0.0394*
COPap_IMF1_RMS	0.4774 ± 0.2280	0.7716 ± 0.5024	0.0395*

* p < 0.05, ** p < 0.01, *** p < 0.001.

To construct a parsimonious and efficient diagnostic model, the Sequential Forward Floating Selection (SFFS) algorithm was employed to identify core biomechanical biomarkers, with the objective of maximizing the subject-level AUC. This process ultimately retained a subset of three core features: the Bin-Entropy of the first IMF in the anterior-posterior direction (COPap_IMF1_BinEntropy), the Standard Deviation of the first IMF in the medio-lateral direction (COPml_IMF1_STD), and the Total Power of the second IMF in the anterior-posterior direction (COPap_IMF2_TotalPower).

Based on these three features, the performance of five machine learning models was evaluated (Table 4), with optimal hyperparameter configurations detailed in Table 5. In terms of overall discriminatory capability, the DT demonstrated superior performance, achieving a mean AUC of 0.9606 ± 0.0371 and a 95% CI of [0.8718, 1.0000], reflecting exceptional stability. RF followed closely with a mean AUC of 0.9179 ± 0.0630 (95% CI [0.7756, 1.0000]), showing no statistically significant difference compared to DT (p = 0.2520). The XGB model yielded a mean AUC of 0.8970 ± 0.0656, which also did not differ significantly from RF (p = 0.5850). In contrast, LR and SVM exhibited significantly lower AUCs (LR: 0.7698 ± 0.1010, p = 0.0236; SVM: 0.7055 ± 0.1062, p = 0.0045).

**TABLE 4:** Comparative performance metrics and statistical validation of five machine learning models for fall risk classification.

Model	AUC	95% CI	Sens	Spec	Th	P-value
DT	0.9606 ± 0.0371	[0.8718-1.0000]	0.8647	0.9150	0.46	0.2520
RF	0.9179 ± 0.0630	[0.7756-1.0000]	0.8647	0.9150	0.44	—
XGB	0.8970 ± 0.0656	[0.7405-1.0000]	1.0000	0.6649	0.29	0.5850
LR	0.7698 ± 0.1010	[0.5469-0.9364]	0.7361	0.7918	0.53	0.0236*
SVM	0.7055 ± 0.1062	[0.4910-0.8955]	0.7361	0.7918	0.29	0.0045**

Sens: Sensitivity; Spec: Specificity; Th: Optimal classification threshold. * p < 0.05, ** p < 0.01 vs. baseline RF, model.

**TABLE 5:** Optimal hyperparameter configurations for the five machine learning classification models.

Model	Hyperparameter	Value
DT	max_depth	3
	min_samples_leaf	1
	class_weight	Balanced
	random_state	42
RF	n_estimators	100
	max_depth	3
	class_weight	Balanced
	random_state	42
XGB	scale_pos_weight	3
	n_estimators	50
	max_depth	2
	learning_rate	0.1
	random_state	42
LR	C	5.0
	class_weight	Balanced
	random_state	42
SVM	C	1.5
	Kernel	Linear
	class_weight	Balanced
	Probability	True
	random_state	42

Regarding the balance of classification metrics, both DT and RF demonstrated highly balanced sensitivity (0.8647) and specificity (0.9150) at their respective optimal thresholds (0.46 and 0.44). This balance indicated their ability to effectively identify Fall Risk subjects while minimizing false positives. Although XGB achieved a sensitivity of 1.0000, its specificity dropped to 0.6649, suggesting a bias towards the positive class. LR and SVM showed moderate balance (Sensitivity: 0.7361; Specificity: 0.7918) but underperformed compared to the tree-based models.

**FIGURE 1:**
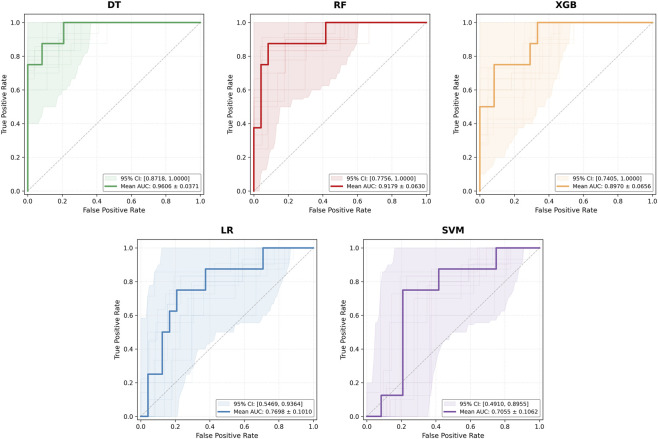
Comparison of ROC curves for the five machine learning models under the rs ec condition.

**FIGURE 2:**
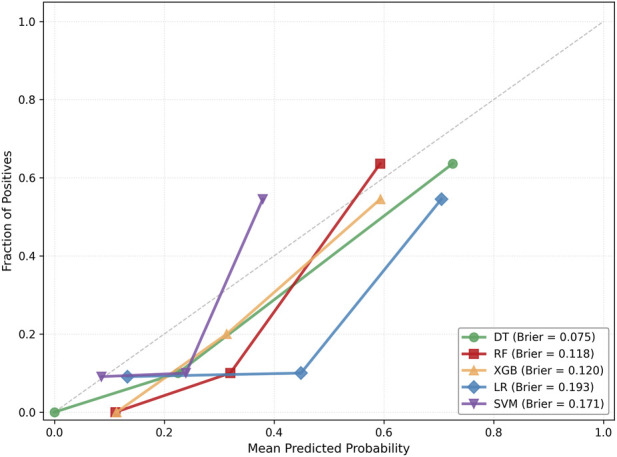
Calibration plots of the five machine learning models under the rs ec condition.

The ROC curves and their corresponding 95% CIs for each model are illustrated in Figure 1. The curves for the tree-based models (DT, RF, XGB) closely approached the top-left corner, maintaining high true positive rates across varying false positive rates, which confirmed their superior discriminatory power. Specifically, DT exhibited the narrowest confidence interval, indicating the highest stability. The curves for RF and XGB were morphologically similar but presented slightly wider confidence intervals, reflecting a degree of variability. Conversely, the curves for the linear models (LR, SVM) were closer to the diagonal line, indicating relatively weaker discriminatory capacity, consistent with the AUC analysis. Figure 2 presents the calibration plots for the five models, illustrating the reliability of their probability outputs across the full range of decision thresholds. Compared to the linear models (LR, SVM), the tree-based models (DT, RF, XGB) achieved superior calibration. Among them, the DT model delivered the most consistent performance, as evidenced by its minimal Brier score (0.075).

**FIGURE 3:**
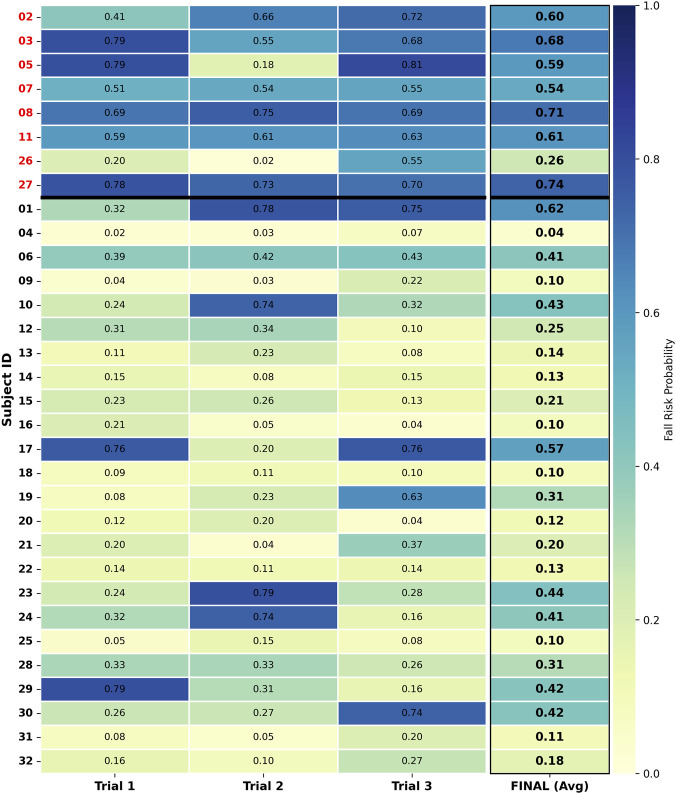
Subject-level heatmap of fall risk probabilities predicted by the Random Forest model.

To further validate individual-level predictive performance, a subject-level fall risk probability heatmap was generated using the RF model (Figure 3). The Fall Risk group (labeled with red IDs) is positioned in the upper section, separated from the No Fall Risk group by a bold black line. The color gradient from light yellow to dark blue represents the fall risk probability (0.0–1.0). Values within the cells indicate the risk probability for corresponding trials or the aggregated result, while the FINAL column represents the mean probability across the three trials for each subject.

In the Fall Risk group, the aggregated probabilities (FINAL) for most subjects exceeded the optimal classification threshold of 0.44. For instance, subjects 03 (0.68), 08 (0.71), and 27 (0.74) maintained high probability levels. Only Subject 26 presented an aggregated probability (0.26) below the threshold, resulting in a misclassification as No Fall Risk (False Negative). In the No Fall Risk group (labeled with black IDs), the majority of subjects exhibited aggregated probabilities below 0.44 and were correctly identified. Although certain subjects showed elevated risk probabilities in single trials, the aggregation strategy of averaging probabilities across three trials successfully corrected these transient fluctuations. Consequently, their FINAL probabilities fell below the 0.44 threshold, ensuring correct classification. Only subjects 01 (Trial 2: 0.78; Trial 3: 0.75) and 17 (Trial 1: 0.76; Trial 3: 0.76) exhibited elevated probabilities in two out of three trials, resulting in aggregated probabilities (Subject 01: 0.62; Subject 17: 0.57) that exceeded the threshold. These constituted the only two cases of misclassification (False Positives) within the No Fall Risk group.

## 4 Discussion

This study proposed and validated an analytical framework for classifying fall risk in patients with PD by integrating EMD with machine learning algorithms. Through a systematic comparison of five standing conditions, 130 multi-scale features, and five mainstream machine learning models, the study identified the rs ec condition as optimal for fall risk discrimination. Furthermore, a parsimonious combination of three core features, selected via EMD decomposition and the SFFS algorithm, combined with tree-based models, achieved accurate fall risk classification. This framework provides an objective, efficient, and clinically translatable tool for early fall risk screening in PD patients.

A key finding of this study is that the rs ec condition yielded superior discriminatory performance compared to other standing conditions, achieving a mean AUC of 0.8244. This finding aligns with the pathophysiological characteristics of PD. As a basal ganglia disorder, the loss of dopaminergic neurons in PD induces sensorimotor integration deficits, leading to postural control abnormalities and balance impairment (Horak, 2006; Frazzitta et al., 2009). Patients often exhibit proprioceptive and vestibular dysfunction, with compromised intrinsic motor rhythm and control. Consequently, they rely heavily on external visual input to compensate for balance deficits. When visual input is removed, this compensatory mechanism is eliminated, significantly exacerbating postural instability (Horak, 2006; Palakurthi and Burugupally, 2019). While unstable surface conditions challenge balance function, they introduce excessive environmental variability, leading to unstable discriminatory performance in this study. In contrast, the rs ec condition offers a standardized, low-variability assessment scenario that accurately reflects the intrinsic postural control capacity of PD patients free from visual compensation, making it an ideal scenario for fall risk assessment (Horak, 2006; Palakurthi and Burugupally, 2019).

Based on the optimal rs ec condition, EMD was employed to decompose the Center of Pressure (COP) signals into multi-scale components. The SFFS algorithm was then utilized to select three core features, achieving minimalist modeling while retaining critical information for fall risk classification. EMD, as an adaptive time-frequency analysis method, decomposes non-linear and non-stationary COP signals into Intrinsic Mode Functions (IMFs) of varying frequency bands without requiring a predetermined basis function (Rilling et al., 2003). The selected feature subset comprised the Bin-Entropy of the first IMF in the anterior-posterior direction (COPap_IMF1_BinEntropy), the Standard Deviation of the first IMF in the medio-lateral direction (COPml_IMF1_STD), and the Total Power of the second IMF in the anterior-posterior direction (COPap_IMF2_TotalPower).

The COPap_IMF1_BinEntropy feature combines high-frequency temporal resolution with non-linear complexity assessment. From a signal processing perspective, IMF1 captures the highest-frequency, smallest-scale fluctuation components of the COP signal. Previous studies have confirmed that dynamic features in higher frequency bands of COP signals exhibit stronger discriminatory power in distinguishing fallers from non-fallers compared to low-frequency components (Fino et al., 2016). Additionally, COP entropy features in the AP direction have been shown to be more sensitive to fall risk in the elderly than those in the ML direction (Montesinos et al., 2018). The introduction of Bin-Entropy quantifies the complexity of the signal state distribution (Fino et al., 2016), capturing the abnormal distribution patterns of postural sway in PD patients within specific frequency bands. The COPml_IMF1_STD feature provides time-domain information on high-frequency fluctuations in the ML direction. Research has indicated that applying EMD to COP signals (both AP and ML) and extracting time-domain statistical parameters from IMFs can enhance the accuracy and sensitivity of PD classification and early detection (Fadil et al., 2021; Safi et al., 2022). In time-series analysis, standard deviation objectively reflects the dispersion and magnitude of signal fluctuations. The inclusion of COPml_IMF1_STD suggests that when PD patients lose visual compensation under eyes-closed conditions, specific changes occur not only in the complexity of postural regulation in the anterior-posterior direction but also in the magnitude of high-frequency fluctuations in the lateral (ML) direction. Finally, COPap_IMF2_TotalPower measures the energy intensity of the sub-high-frequency component in the AP direction. Its selection implies potential abnormal patterns in the energy distribution of postural control among PD patients with high fall risk. By combining this frequency-domain feature with the aforementioned time-domain (STD) and non-linear (Bin-Entropy) features, complementary discriminatory information is provided across energy, magnitude, and regularity dimensions, thereby enabling the model to comprehensively identify fall risk patterns in PD. Compared to existing PD fall risk prediction models, which report optimal AUCs between 0.71 and 0.79 (Malaguti et al., 2025), the present study achieved a subject-level AUC of up to 0.96 using only three EMD-derived features. This suggests that features based on high-frequency COP components may more precisely capture subtle, concealed postural control deficits.

The performance comparison of classification algorithms revealed that tree-based models (DT, RF, XGB) generally outperformed linear models (LR, SVM). Statistical tests confirmed that the baseline RF model significantly outperformed LR (p = 0.0236) and SVM (p = 0.0045). This quantitatively demonstrates that the biomechanical representation of fall risk in PD is not a simple linear superposition but rather a complex interaction between features of different frequency bands and dimensions. RF and other ensemble algorithms, through their collaborative decision-tree mechanisms, effectively capture these non-linear decision boundaries and exhibit strong robustness in small-sample scenarios. Notably, although the DT achieved the highest numerical AUC, and XGB also demonstrated a high discriminatory level, their performance did not differ significantly from RF (p > 0.05). This result indicates that within the current feature space, the capture of fall risk by tree-based algorithms has approached saturation, and minor numerical differences between algorithms likely stem from variations in internal inductive bias rather than fundamental differences in discriminatory efficacy. Furthermore, regarding the balance required for clinical screening, XGB showed extremely high sensitivity but lower specificity, suggesting a potential bias towards the positive class in small samples. In contrast, RF and DT exhibited a better balance between sensitivity and specificity, making tree-based models processing EMD-derived multi-scale biomechanical signals more suitable for clinical applications than traditional linear algorithms.

Subject-level heatmaps (Figure 3) revealed fluctuations in performance across different trials for the same subject. The observed probability fluctuations in single trials may be related to the instability of motor symptoms in PD patients. Addressing this characteristic, the multi-trial probability aggregation strategy adopted in this study enhanced assessment robustness. Results confirmed that averaging probabilities across three trials effectively smoothed out misclassifications caused by single-trial fluctuations, bringing the subject’s final risk classification closer to the true label.

This study has several limitations. First, the sample size is relatively small. Although LOSO-CV and Bootstrap resampling were employed to ensure statistical reliability, the model’s generalization capability requires further verification in larger, multi-center clinical cohorts. Second, as this study utilized data exclusively from subjects in the off-medication state, the model’s current applicability is primarily limited to assessing postural control after excluding medication effects. Finally, the Mini-BESTest score was utilized as a surrogate label for fall risk. While this scale possesses high clinical authority, the use of a fixed cutoff of 21 represents a limitation, as literature-reported thresholds for discriminating fall risk in PD typically vary between 16 and 26. Consequently, the diagnostic consistency of the model across this continuous spectrum of thresholds was not fully evaluated in the present study. Future research should, therefore, validate the model’s robustness across diverse cutoffs in larger cohorts, while also incorporating prospective longitudinal follow-up data to establish its predictive validity in real-world scenarios. Additionally, recent reviews have highlighted that extracting biomarkers using sensors is a growing trend in identifying PD fall risk (Bradley et al., 2025). Future research could integrate the proposed framework to further validate the assessment value of this feature subset under multi-source sensor monitoring.

## 5 Conclusion

This study constructed and validated an analytical framework for classifying fall risk in patients with PD based on EMD multi-scale features and machine learning algorithms. The results confirmed that among all tested scenarios, the rs ec condition yielded the optimal efficacy for identifying fall risk. Experiments demonstrated that a parsimonious feature subset comprising COPap_IMF1_BinEntropy, COPml_IMF1_STD, and COPap_IMF2_TotalPower, when combined with machine learning algorithms, achieved high-precision risk classification. Among the five models compared, tree-based models—specifically DT and RF—exhibited superior and balanced discriminatory performance. Furthermore, the multi-trial probability aggregation strategy adopted in this study effectively stabilized subject performance fluctuations, enhancing the robustness of the classification results. In summary, this study proposes an objective and efficient paradigm for PD fall risk classification, providing methodological support for early clinical screening. Future research will further verify the model’s generalization capability in large-scale datasets and evaluate its classification performance across different disease stages and medication states.

## Data Availability

Publicly available datasets were analyzed in this study. The data can be found in the Figshare repository: https://doi.org/10.6084/m9.figshare.13530587. Citation: de Oliveira, C. E. N. et al. (2022). A public data set with ground reaction forces of human balance in individuals with Parkinson’s disease.

## References

[B1] Allen N. E. Schwarzel A. K. Canning C. G. (2013). Recurrent falls in Parkinson’s disease: a systematic review. Parkinson’s Disease 2013, 906274. 10.1155/2013/906274 23533953 PMC3606768

[B2] Błaszczyk J. W. (2016). The use of force-plate posturography in the assessment of postural instability. Gait & Posture 44, 1–6. 10.1016/j.gaitpost.2015.10.014 27004624

[B3] Bloem B. R. Hausdorff J. M. Visser J. E. Giladi N. (2004). Falls and freezing of gait in Parkinson’s disease: a review of two interconnected, episodic phenomena. Mov. Disorders Official Journal Mov. Disord. Soc. 19, 871–884. 10.1002/mds.20115 15300651

[B4] Bradley M. O’Loughlin S. Donlon E. Gallagher A. O’Keeffe C. Inocentes J. (2025). Determining falls risk in people with parkinson’s disease using wearable sensors: a systematic review. Sensors 25, 4071. 10.3390/s25134071 40648326 PMC12251786

[B5] Chen B. Liu P. Xiao F. Liu Z. Wang Y. (2021). Review of the upright balance assessment based on the force plate. Int. Journal Environmental Research Public Health 18, 2696. 10.3390/ijerph18052696 33800119 PMC7967421

[B6] Contreras A. Grandas F. (2012). Risk of falls in parkinson’s disease: a cross-sectional study of 160 patients. Parkinson’s Dis. 2012, 362572. 10.1155/2012/362572 22292126 PMC3265111

[B7] De Oliveira C. E. N. Ribeiro de Souza C. Treza R. de C. Hondo S. M. Los Angeles E. Bernardo C. (2022). A public data set with ground reaction forces of human balance in individuals with parkinson’s disease. Front. Neuroscience 16, 865882. 10.3389/fnins.2022.865882 35516808 PMC9063313

[B8] Dorsey E. R. Elbaz A. Nichols E. Abbasi N. Abd-Allah F. Abdelalim A. (2018). Global, regional, and national burden of Parkinson’s disease, 1990–2016: a systematic analysis for the global burden of disease study 2016. Lancet Neurology 17, 939–953. 10.1016/S1474-4422(18)30295-3 30287051 PMC6191528

[B9] Duarte M. Freitas S. M. (2010). Revision of posturography based on force plate for balance evaluation. Braz. J. Physical Therapy 14, 183–192. 10.1590/S1413-35552010000300003 20730361

[B10] Duncan R. P. Leddy A. L. Cavanaugh J. T. Dibble L. E. Ellis T. D. Ford M. P. (2013). Comparative utility of the BESTest, mini-BESTest, and brief-BESTest for predicting falls in individuals with parkinson disease: a cohort study. Phys. Therapy 93, 542–550. 10.2522/ptj.20120302 23174567 PMC3613340

[B11] Efron B. Tibshirani R. J. (1994). An introduction to the bootstrap. Chapman and Hall/CRC. Available online at: https://api.taylorfrancis.com/content/books/mono/download?identifierName=doi&identifierValue=10.1201/9780429246593&type=googlepdf (Accessed February 21, 2026).

[B12] Fadil R. Huether A. Brunnemer R. Blaber A. P. Lou J.-S. Tavakolian K. (2021). “Early detection of Parkinson’s disease using center of pressure data and machine learning,” in 2021 43rd Annual International Conference of the IEEE Engineering in Medicine & Biology Society (EMBC) (IEEE), 2433–2436. 10.1109/EMBC46164.2021.9630451 34891772

[B13] Fasano A. Canning C. G. Hausdorff J. M. Lord S. Rochester L. (2017). Falls in Parkinson’s disease: a complex and evolving picture. Mov. Disorders 32, 1524–1536. 10.1002/mds.27195 29067726

[B14] Fino P. C. Mojdehi A. R. Adjerid K. Habibi M. Lockhart T. E. Ross S. D. (2016). Comparing postural stability entropy analyses to differentiate fallers and non-fallers. Ann. Biomed. Eng. 44, 1636–1645. 10.1007/s10439-015-1479-0 26464267 PMC4833705

[B15] Frazzitta G. Maestri R. Uccellini D. Bertotti G. Abelli P. (2009). Rehabilitation treatment of gait in patients with Parkinson’s disease with freezing: a comparison between two physical therapy protocols using visual and auditory cues with or without treadmill training. Mov. Disord. 24, 1139–1143. 10.1002/mds.22491 19370729

[B16] Gao C. Sun H. Wang T. Tang M. Bohnen N. I. Müller M. L. (2018). Model-based and model-free machine learning techniques for diagnostic prediction and classification of clinical outcomes in Parkinson’s disease. Sci. Reports 8, 7129. 10.1038/s41598-018-24783-4 29740058 PMC5940671

[B17] Goetz C. G. Tilley B. C. Shaftman S. R. Stebbins G. T. Fahn S. Martinez-Martin P. (2008). Movement disorder Society-sponsored revision of the unified Parkinson’s disease rating scale (MDS-UPDRS): scale presentation and clinimetric testing results. Mov. Disorders Official Journal Mov. Disord. Soc. 23, 2129–2170. 10.1002/mds.22340 19025984

[B18] Hely M. A. Morris J. G. Reid W. G. Trafficante R. (2005). Sydney multicenter study of Parkinson’s disease: non-l-dopa–responsive problems dominate at 15 years. Mov. Disorders Official Journal Mov. Disord. Soc. 20, 190–199. 10.1002/mds.20324 15551331

[B19] Hoehn M. M. Yahr M. D. (1967). Parkinsonism: onset, progression, and mortality. Neurology 17, 427. 10.1212/WNL.17.5.427 6067254

[B20] Horak F. B. (2006). Postural orientation and equilibrium: what do we need to know about neural control of balance to prevent falls? Age Ageing 35, ii7–ii11. 10.1093/ageing/afl077 16926210

[B21] Huang N. E. Shen Z. Long S. R. Wu M. C. Shih H. H. Zheng Q. (1998). The empirical mode decomposition and the hilbert spectrum for nonlinear and non-stationary time series analysis. Proc. R. Soc. Lond. Ser. A Mathematical, Physical Engineering Sciences 454, 903–995. 10.1098/rspa.1998.0193

[B22] Hubble R. P. Naughton G. A. Silburn P. A. Cole M. H. (2015). Wearable sensor use for assessing standing balance and walking stability in people with Parkinson’s disease: a systematic review. PloS One 10, e0123705. 10.1371/journal.pone.0123705 25894561 PMC4403989

[B23] Jankovic J. (2008). Parkinson’s disease: clinical features and diagnosis. J. Neurology, Neurosurgery & Psychiatry 79, 368–376. 10.1136/jnnp.2007.131045 18344392

[B24] Kowal S. L. Dall T. M. Chakrabarti R. Storm M. V. Jain A. (2013). The current and projected economic burden of Parkinson’s disease in the United States. Mov. Disord. 28, 311–318. 10.1002/mds.25292 23436720

[B25] Leddy A. L. Crowner B. E. Earhart G. M. (2011). Utility of the Mini-BESTest, BESTest, and BESTest sections for balance assessments in individuals with parkinson disease. J. Neurologic Physical Therapy 35, 90–97. 10.1097/NPT.0b013e31821a620c 21934364 PMC3178037

[B26] Malaguti M. C. Longo C. Moroni M. Ragni F. Bovo S. Chierici M. (2025). Machine learning predicts risk of falls in parkison’s disease patients in a multicenter observational study. Eur. J. Neurology 32, e70118. 10.1111/ene.70118 40304115 PMC12041891

[B27] Mancini M. Horak F. B. (2010). The relevance of clinical balance assessment tools to differentiate balance deficits. Eur. Journal Physical Rehabilitation Medicine 46, 239–248. 20485226 PMC3033730

[B28] Montesinos L. Castaldo R. Pecchia L. (2018). On the use of approximate entropy and sample entropy with centre of pressure time-series. J. NeuroEngineering Rehabilitation 15, 116. 10.1186/s12984-018-0465-9 30541587 PMC6291990

[B29] Moon S. Song H.-J. Sharma V. D. Lyons K. E. Pahwa R. Akinwuntan A. E. (2020). Classification of Parkinson’s disease and essential tremor based on balance and gait characteristics from wearable motion sensors *via* machine learning techniques: a data-driven approach. J. Neuroengineering Rehabilitation 17, 125. 10.1186/s12984-020-00756-5 32917244 PMC7488406

[B30] Pachori R. B. Hewson D. Snoussi H. Duchêne J. (2008). “Analysis of center of pressure signals using empirical mode decomposition and fourier-bessel expansion,” in TENCON 2008-2008 IEEE Region 10 Conference (IEEE), 1–6.

[B31] Palakurthi B. Burugupally S. P. (2019). Postural instability in parkinson’s disease: a review. Brain Sci. 9, 239. 10.3390/brainsci9090239 31540441 PMC6770017

[B32] Palmieri R. M. Ingersoll C. D. Stone M. B. Krause B. A. (2002). Center-of-pressure parameters used in the assessment of postural control. J. Sport Rehabilitation 11, 51–66. 10.1123/jsr.11.1.51

[B33] Pickering R. M. Grimbergen Y. A. Rigney U. Ashburn A. Mazibrada G. Wood B. (2007). A meta-analysis of six prospective studies of falling in Parkinson’s disease. Mov. Disord. 22, 1892–1900. 10.1002/mds.21598 17588236

[B34] Pincus S. M. (1991). Approximate entropy as a measure of system complexity. Proc. National Academy Sciences 88, 2297–2301. 10.1073/pnas.88.6.2297 11607165 PMC51218

[B35] Poewe W. Seppi K. Tanner C. M. Halliday G. M. Brundin P. Volkmann J. (2017). Parkinson disease. Nat. Reviews Dis. Primers 3, 1–21. 10.1038/nrdp.2017.13 28332488

[B36] Pouwels S. Bazelier M. De Boer A. Weber W. J. Neef C. Cooper C. (2013). Risk of fracture in patients with Parkinson’s disease. Osteoporos. Int. 24, 2283–2290. 10.1007/s00198-013-2300-2 23430103

[B37] Prieto T. E. Myklebust J. B. Hoffmann R. G. Lovett E. G. Myklebust B. M. (1996). Measures of postural steadiness: differences between healthy young and elderly adults. IEEE Trans. Biomedical Engineering 43, 956–966. 10.1109/10.532130 9214811

[B38] Pudil P. Novovičová J. Kittler J. (1994). Floating search methods in feature selection. Pattern Recognition Letters 15, 1119–1125. 10.1016/0167-8655(94)90127-9

[B39] Richman J. S. Moorman J. R. (2000). Physiological time-series analysis using approximate entropy and sample entropy. Am. Journal Physiology-Heart Circulatory Physiology 278, H2039–H2049. 10.1152/ajpheart.2000.278.6.H2039 10843903

[B40] Rilling G. Flandrin P. Goncalves P. (2003). “On empirical mode decomposition and its algorithms,” in IEEE-EURASIP workshop on nonlinear signal and image processing NSIP-03.

[B41] Rufibach K. (2010). Use of brier score to assess binary predictions. J. Clinical Epidemiology 63, 938–939. 10.1016/j.jclinepi.2009.11.009 20189763

[B42] Saeb S. Lonini L. Jayaraman A. Mohr D. C. Kording K. P. (2017). The need to approximate the use-case in clinical machine learning. Gigascience 6, gix019–gix019. 10.1093/gigascience/gix019 28327985 PMC5441397

[B43] Safi K. Aly W. H. F. AlAkkoumi M. Kanj H. Ghedira M. Hutin E. (2022). EMD-Based method for supervised classification of Parkinson’s disease patients using balance control data. Bioengineering 9, 283. 10.3390/bioengineering9070283 35877334 PMC9311556

[B44] Sotirakis C. Brzezicki M. A. Patel S. Conway N. FitzGerald J. J. Antoniades C. A. (2024). Predicting future fallers in Parkinson’s disease using kinematic data over a period of 5 years. Npj Digit. Med. 7, 345. 10.1038/s41746-024-01311-5 39638907 PMC11621420

[B45] Van Calster B. McLernon D. J. van Smeden M. Wynants L. Steyerberg E. W. Bossuyt P. (2019). Calibration: the achilles heel of predictive analytics. BMC Med. 17, 230. 10.1186/s12916-019-1466-7 31842878 PMC6912996

[B46] Williams D. R. Watt H. C. Lees A. J. (2006). Predictors of falls and fractures in bradykinetic rigid syndromes: a retrospective study. J. Neurology, Neurosurg. & Psychiatry 77, 468–473. 10.1136/jnnp.2005.074070 16543524 PMC2077491

[B47] Winser S. J. Kannan P. Bello U. M. Whitney S. L. (2019). Measures of balance and falls risk prediction in people with Parkinson’s disease: a systematic review of psychometric properties. Clin. Rehabilitation 33, 1949–1962. 10.1177/0269215519877498 31571503 PMC6826874

[B48] Winter D. A. (1995). Human balance and posture control during standing and walking. Gait & Posture 3, 193–214. 10.1016/0966-6362(96)82849-9

[B49] Wood B. Bilclough J. Bowron A. Walker R. (2002). Incidence and prediction of falls in Parkinson’s disease: a prospective multidisciplinary study. J. Neurology, Neurosurg. & Psychiatry 72, 721–725. 10.1136/jnnp.72.6.721 12023412 PMC1737913

[B50] Yardley L. Beyer N. Hauer K. Kempen G. Piot-Ziegler C. Todd C. (2005). Development and initial validation of the falls efficacy scale-international (FES-I). Age Ageing 34, 614–619. 10.1093/ageing/afi196 16267188

[B51] Youden W. J. (1950). Index for rating diagnostic tests. Cancer 3, 32–35. 10.1002/1097-0142(1950)3:1<32::AID-CNCR2820030106>3.0.CO 15405679

[B52] Zeng W. Yuan C. Wang Q. Liu F. Wang Y. (2019). Classification of gait patterns between patients with Parkinson’s disease and healthy controls using phase space reconstruction (PSR), empirical mode decomposition (EMD) and neural networks. Neural Netw. 111, 64–76. 10.1016/j.neunet.2018.12.012 30690285

